# 2-Amino-4-*tert*-butyl-5-(4-chloro­benz­yl)thia­zole

**DOI:** 10.1107/S160053680803715X

**Published:** 2008-11-13

**Authors:** Yong-Tao Wang, Lin Xia, Ai-Xi Hu, Gao Cao, Juan-Juan Xu

**Affiliations:** a College of Chemistry and Chemical Engineering, Hunan University 410082, Changsha, People’s Republic of China

## Abstract

In the title compound, C_14_H_17_ClN_2_S, the dihedral angle between the planes of the thia­zole and chloro­phenyl rings is 88.86 (3)°. In the crystal, inversion dimers occur, linked by pairs of N—H⋯N hydrogen bonds.

## Related literature

For background on 2-amino-4-aryl­thia­zoles and their wide-ranging anti­fungal activities, see: Hu *et al.* (2007*a* ▶); Marcantonio *et al.* (2002 ▶). For related structures, see: Cao *et al.* (2007 ▶); He *et al.* (2006 ▶); Hu *et al.* (2007*b* ▶); Xu *et al.* (2007 ▶).
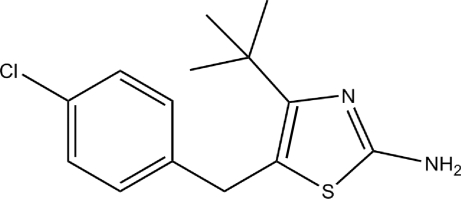


## Experimental

### 

#### Crystal data

C_14_H_17_ClN_2_S
*M* *_r_* = 280.81
Monoclinic, 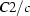

*a* = 21.1775 (13) Å
*b* = 5.8544 (4) Å
*c* = 22.8193 (14) Å
β = 98.5480 (10)°
*V* = 2797.7 (3) Å^3^
*Z* = 8
Mo *K*α radiation
μ = 0.41 mm^−1^
*T* = 173 (2) K
0.48 × 0.29 × 0.17 mm

#### Data collection

Bruker SMART 1000 CCD diffractometer
Absorption correction: multi-scan (*SADABS*; Sheldrick, 2004 ▶) *T*_min_ = 0.829, *T*_max_ = 0.934
6230 measured reflections
2705 independent reflections
2187 reflections with *I* > 2σ(*I*)
*R*_int_ = 0.021

#### Refinement

*R*[*F* ^2^ > 2σ(*F* ^2^)] = 0.039
*wR*(*F* ^2^) = 0.120
*S* = 1.06
2705 reflections
166 parameters
H-atom parameters constrained
Δρ_max_ = 0.32 e Å^−3^
Δρ_min_ = −0.32 e Å^−3^

### 

Data collection: *SMART* (Bruker, 2001 ▶); cell refinement: *SAINT-Plus* (Bruker, 2003 ▶); data reduction: *SAINT-Plus*; program(s) used to solve structure: *SHELXTL* (Sheldrick, 2008 ▶); program(s) used to refine structure: *SHELXTL*; molecular graphics: *SHELXTL*; software used to prepare material for publication: *SHELXTL*.

## Supplementary Material

Crystal structure: contains datablocks I, global. DOI: 10.1107/S160053680803715X/sg2282sup1.cif

Structure factors: contains datablocks I. DOI: 10.1107/S160053680803715X/sg2282Isup2.hkl

Additional supplementary materials:  crystallographic information; 3D view; checkCIF report

## Figures and Tables

**Table 1:** Hydrogen-bond geometry (Å, °)

*D*—H⋯*A*	*D*—H	H⋯*A*	*D*⋯*A*	*D*—H⋯*A*
N2—H2*A*⋯N1^i^	0.88	2.24	3.032 (2)	150

Symmetry code: (i) 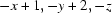.

**Fig. 1.**
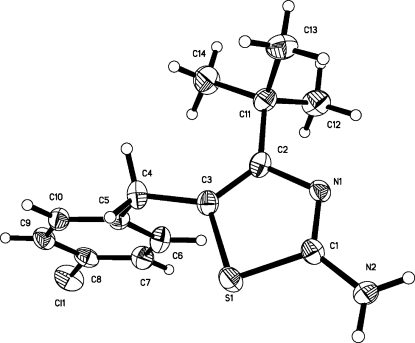
Molecular structure showing 30% probability displacement ellipsoids.H atoms are drawn as spheres of arbitrary radii. Only the major occupied sites of the disordered tert-butyl group are showen.

**Fig. 2.**
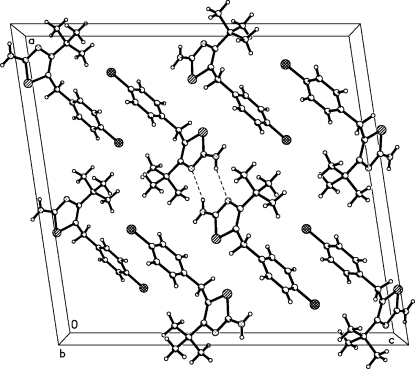
Packing diagram showing the N—H···N hydrogen bonds.

## supplementary crystallographic information

### Comment

2-Amino-4-arylthiazoles are an important class of heterocyclic compounds in the field of organic pharmaceutial chenistry (Hu *et al.*, 2007a, Marcantonio *et al.*,2002). Because of their wide-ranging antifungal activities, The structure of 2-amino-4-arylthiazoles were reported before (Cao,*et al.*,2007, He *et al.*,2006, Hu *et al.*,2007b, Xu, *et al.*,2007). Herein we report the synthesis and crystal structure of 2-amino-4-*tert*-butyl-5-(4- chlorobenzyl)thiazole(I). The dihedral angle between the planes of thiazole and the chlorophenyl ring is 88.86 °. The molecules are linked by N—H···N hydrogen bonds.

### Experimental

0.01 mol of 1-(4-Chlorophenyl)-4,4-dimethylpentan-3-one was dissolved in 100 ml e thanol and the mixture was stirred and heated to reflex. 0.012 mol of cupric chloride was added by dropwise. The reaction was monitored by TLC, after it finished, filtered the mixture and concentrated *in vacuo*. The residue was taken up in dichloromethane, washed with 10% hydrochloric acid, then washed with water until the solution was neutral, dried and concentrated *in vacuo* to give 4-chloro-1-(4-chlorophenyl)-4,4-dimethylpentan -3-one, yield 87%. Then a solution with 0.005 mol of thiurea and 0.005 mol of 4-chloro-1-(4-chlorophenyl)-4,4-dimethylpentan -3-one in 50 ml of ethanol was refluxed for 10 h. After finishing the reaction, added 10 ml ammonia and continus to stir the sulution 2 h. Then the solution was cooled and the precipitate formed was filtered out, dried, giving white crystals of title compound,yield 73.8%. The crystals for X-ray structure determination were obtained by slow evaporation of an ethanol solution at room temperature.

### Refinement

Methyl H atoms were placed in calculated positions, with C—H = 0.96 Å, and torsion angles were refined, with *U*_iso_(H) = 1.5Ueq(C). Other H atoms were placed in geometrically idealized positions and refined as riding model, with N—H distance of 0.86 Å, C—H distances of 0.98Å (C3—H3), 0.93Å (aromatic H atoms) and 0.97Å (methylene H atoms). The constraint *U*_iso_(H) = 1.2Ueq(carrier) was applied.

### Crystal data

**Table:**

C_14_H_17_ClN_2_S	*F*_000_ = 1184
*M**_r_* = 280.81	*D*_x_ = 1.333 Mg m^−^^3^
Monoclinic, *C*2/*c*	Melting point: 390 K
Hall symbol: -C 2yc	Mo *K*α radiation λ = 0.71073 Å
*a* = 21.1775 (13) Å	Cell parameters from 3521 reflections
*b* = 5.8544 (4) Å	θ = 2.8–26.9º
*c* = 22.8193 (14) Å	µ = 0.41 mm^−^^1^
β = 98.5480 (10)º	*T* = 173 (2) K
*V* = 2797.7 (3) Å^3^	Block, colourless
*Z* = 8	0.48 × 0.29 × 0.17 mm

### Data collection

**Table:**

Bruker SMART 1000 CCD diffractometer	2705 independent reflections
Radiation source: fine-focus sealed tube	2187 reflections with *I* > 2σ(*I*)
Monochromator: graphite	*R*_int_ = 0.022
*T* = 173(2) K	θ_max_ = 26.0º
ω scans	θ_min_ = 1.8º
Absorption correction: multi-scan(SADABS; Sheldrick, 2004)	*h* = −26→23
*T*_min_ = 0.829, *T*_max_ = 0.934	*k* = −5→7
6230 measured reflections	*l* = −19→28

### Refinement

**Table:**

Refinement on *F*^2^	Secondary atom site location: difference Fourier map
Least-squares matrix: full	Hydrogen site location: inferred from neighbouring sites
*R*[*F*^2^ > 2σ(*F*^2^)] = 0.039	H-atom parameters constrained
*wR*(*F*^2^) = 0.120	*w* = 1/[σ^2^(*F*_o_^2^) + (0.0686*P*)^2^ + 2.6543*P*] where *P* = (*F*_o_^2^ + 2*F*_c_^2^)/3
*S* = 1.06	(Δ/σ)_max_ = 0.001
2705 reflections	Δρ_max_ = 0.32 e Å^−^^3^
166 parameters	Δρ_min_ = −0.32 e Å^−^^3^
Primary atom site location: structure-invariant direct methods	Extinction correction: none

### Special details

**Table:**

Experimental. ^1^H NMR (CDCl_3_, 400 MHz) (ppm):1.32(s,9H,3CH_3_),4.1(s,2H,CH_2_), 4.8(bs,2H,NH_2_),7.12(d,J=8.0 Hz,2H,2,6-C_6_H_4_Cl), 7.26(d,J=8.0Hz,2H,3,5-C_6_H_4_Cl)
Geometry. All e.s.d.'s (except the e.s.d. in the dihedral angle between two l.s. planes) are estimated using the full covariance matrix. The cell e.s.d.'s are taken into account individually in the estimation of e.s.d.'s in distances, angles and torsion angles; correlations between e.s.d.'s in cell parameters are only used when they are defined by crystal symmetry. An approximate (isotropic) treatment of cell e.s.d.'s is used for estimating e.s.d.'s involving l.s. planes.
Refinement. Refinement of *F*^2^ against ALL reflections. The weighted *R*-factor *wR* and goodness of fit *S* are based on *F*^2^, conventional *R*-factors *R* are based on *F*, with *F* set to zero for negative *F*^2^. The threshold expression of *F*^2^ > σ(*F*^2^) is used only for calculating *R*-factors(gt) *etc*. and is not relevant to the choice of reflections for refinement. *R*-factors based on *F*^2^ are statistically about twice as large as those based on *F*, and *R*- factors based on ALL data will be even larger.

### Fractional atomic coordinates and isotropic or equivalent isotropic displacement parameters (Å^2^)

**Table:**

	*x*	*y*	*z*	*U*_iso_*/*U*_eq_	
Cl1	0.13949 (3)	0.57508 (12)	0.24064 (3)	0.0437 (2)	
S1	0.33404 (2)	0.74487 (9)	−0.00369 (2)	0.02787 (18)	
C1	0.40616 (9)	0.8911 (4)	0.00413 (9)	0.0235 (4)	
C2	0.42658 (10)	0.6258 (3)	0.07470 (8)	0.0228 (4)	
C3	0.36579 (10)	0.5622 (3)	0.05422 (9)	0.0244 (4)	
C4	0.32309 (10)	0.3710 (4)	0.06838 (9)	0.0284 (5)	
H4A	0.3504	0.2416	0.0845	0.034*	
H4B	0.2975	0.3188	0.0309	0.034*	
C5	0.27747 (9)	0.4274 (3)	0.11205 (9)	0.0229 (4)	
C6	0.27927 (11)	0.6335 (4)	0.14241 (9)	0.0301 (5)	
H6	0.3101	0.7454	0.1361	0.036*	
C7	0.23707 (11)	0.6786 (4)	0.18159 (10)	0.0309 (5)	
H7	0.2389	0.8202	0.2021	0.037*	
C8	0.19240 (10)	0.5176 (4)	0.19071 (9)	0.0287 (5)	
C9	0.18919 (10)	0.3095 (4)	0.16165 (9)	0.0296 (5)	
H9	0.1587	0.1978	0.1687	0.036*	
C10	0.23148 (10)	0.2676 (4)	0.12204 (9)	0.0267 (5)	
H10	0.2291	0.1266	0.1012	0.032*	
C11	0.47436 (10)	0.5254 (4)	0.12457 (9)	0.0260 (5)	
C12	0.50271 (12)	0.7208 (4)	0.16519 (10)	0.0359 (6)	
H12A	0.4687	0.7939	0.1833	0.054*	
H12B	0.5224	0.8336	0.1418	0.054*	
H12C	0.5351	0.6594	0.1964	0.054*	
C13	0.52788 (11)	0.4074 (4)	0.09748 (10)	0.0358 (6)	
H13A	0.5609	0.3542	0.1292	0.054*	
H13B	0.5467	0.5160	0.0723	0.054*	
H13C	0.5103	0.2768	0.0736	0.054*	
C14	0.44542 (13)	0.3499 (5)	0.16253 (11)	0.0425 (6)	
H14A	0.4077	0.4155	0.1763	0.064*	
H14B	0.4770	0.3087	0.1968	0.064*	
H14C	0.4330	0.2131	0.1388	0.064*	
N1	0.44938 (8)	0.8119 (3)	0.04551 (7)	0.0228 (4)	
N2	0.41428 (9)	1.0743 (3)	−0.03037 (8)	0.0289 (4)	
H2A	0.4509	1.1483	−0.0254	0.035*	
H2B	0.3830	1.1191	−0.0577	0.035*	

### Atomic displacement parameters (Å^2^)

**Table:**

	*U*^11^	*U*^22^	*U*^33^	*U*^12^	*U*^13^	*U*^23^
Cl1	0.0357 (3)	0.0626 (4)	0.0359 (3)	0.0072 (3)	0.0153 (3)	−0.0044 (3)
S1	0.0206 (3)	0.0356 (3)	0.0269 (3)	−0.0032 (2)	0.0018 (2)	0.0018 (2)
C1	0.0205 (10)	0.0295 (11)	0.0213 (10)	−0.0018 (8)	0.0060 (8)	−0.0023 (8)
C2	0.0255 (11)	0.0245 (10)	0.0196 (9)	−0.0025 (8)	0.0076 (8)	−0.0010 (8)
C3	0.0265 (11)	0.0249 (11)	0.0227 (10)	−0.0026 (8)	0.0062 (8)	−0.0009 (8)
C4	0.0279 (11)	0.0287 (11)	0.0297 (11)	−0.0075 (9)	0.0077 (9)	−0.0046 (9)
C5	0.0219 (10)	0.0246 (10)	0.0217 (10)	−0.0012 (8)	0.0016 (8)	0.0023 (8)
C6	0.0337 (12)	0.0275 (11)	0.0293 (11)	−0.0057 (9)	0.0059 (9)	−0.0021 (9)
C7	0.0348 (12)	0.0293 (11)	0.0280 (11)	0.0008 (10)	0.0033 (9)	−0.0067 (9)
C8	0.0246 (11)	0.0404 (12)	0.0215 (10)	0.0054 (9)	0.0044 (8)	0.0026 (9)
C9	0.0246 (11)	0.0363 (12)	0.0280 (11)	−0.0025 (9)	0.0045 (9)	0.0040 (9)
C10	0.0268 (11)	0.0253 (11)	0.0278 (11)	−0.0018 (9)	0.0036 (9)	0.0011 (8)
C11	0.0283 (11)	0.0275 (11)	0.0223 (10)	0.0000 (9)	0.0043 (8)	0.0049 (8)
C12	0.0456 (15)	0.0374 (13)	0.0220 (11)	0.0007 (11)	−0.0043 (10)	−0.0004 (9)
C13	0.0376 (13)	0.0377 (13)	0.0319 (12)	0.0090 (10)	0.0049 (10)	0.0052 (10)
C14	0.0419 (14)	0.0481 (15)	0.0377 (13)	−0.0053 (12)	0.0064 (11)	0.0190 (12)
N1	0.0222 (9)	0.0263 (9)	0.0204 (8)	−0.0008 (7)	0.0044 (7)	0.0030 (7)
N2	0.0248 (9)	0.0323 (10)	0.0285 (9)	−0.0004 (8)	0.0005 (7)	0.0105 (8)

### Geometric parameters (Å, °)

**Table:**

Cl1—C8	1.746 (2)	C8—C9	1.383 (3)
S1—C1	1.737 (2)	C9—C10	1.386 (3)
S1—C3	1.754 (2)	C9—H9	0.9500
C1—N1	1.299 (3)	C10—H10	0.9500
C1—N2	1.356 (3)	C11—C14	1.530 (3)
C2—C3	1.355 (3)	C11—C13	1.534 (3)
C2—N1	1.400 (3)	C11—C12	1.537 (3)
C2—C11	1.524 (3)	C12—H12A	0.9800
C3—C4	1.504 (3)	C12—H12B	0.9800
C4—C5	1.524 (3)	C12—H12C	0.9800
C4—H4A	0.9900	C13—H13A	0.9800
C4—H4B	0.9900	C13—H13B	0.9800
C5—C6	1.389 (3)	C13—H13C	0.9800
C5—C10	1.393 (3)	C14—H14A	0.9800
C6—C7	1.380 (3)	C14—H14B	0.9800
C6—H6	0.9500	C14—H14C	0.9800
C7—C8	1.373 (3)	N2—H2A	0.8800
C7—H7	0.9500	N2—H2B	0.8800
			
C1—S1—C3	89.44 (10)	C9—C10—C5	121.5 (2)
N1—C1—N2	124.57 (19)	C9—C10—H10	119.2
N1—C1—S1	114.38 (15)	C5—C10—H10	119.2
N2—C1—S1	121.04 (15)	C2—C11—C14	113.86 (19)
C3—C2—N1	115.28 (18)	C2—C11—C13	108.74 (17)
C3—C2—C11	130.02 (19)	C14—C11—C13	107.96 (19)
N1—C2—C11	114.70 (17)	C2—C11—C12	108.64 (17)
C2—C3—C4	134.5 (2)	C14—C11—C12	108.16 (18)
C2—C3—S1	109.30 (15)	C13—C11—C12	109.42 (19)
C4—C3—S1	116.10 (15)	C11—C12—H12A	109.5
C3—C4—C5	116.06 (17)	C11—C12—H12B	109.5
C3—C4—H4A	108.3	H12A—C12—H12B	109.5
C5—C4—H4A	108.3	C11—C12—H12C	109.5
C3—C4—H4B	108.3	H12A—C12—H12C	109.5
C5—C4—H4B	108.3	H12B—C12—H12C	109.5
H4A—C4—H4B	107.4	C11—C13—H13A	109.5
C6—C5—C10	118.02 (19)	C11—C13—H13B	109.5
C6—C5—C4	122.77 (18)	H13A—C13—H13B	109.5
C10—C5—C4	119.21 (18)	C11—C13—H13C	109.5
C7—C6—C5	121.1 (2)	H13A—C13—H13C	109.5
C7—C6—H6	119.4	H13B—C13—H13C	109.5
C5—C6—H6	119.4	C11—C14—H14A	109.5
C8—C7—C6	119.6 (2)	C11—C14—H14B	109.5
C8—C7—H7	120.2	H14A—C14—H14B	109.5
C6—C7—H7	120.2	C11—C14—H14C	109.5
C7—C8—C9	121.2 (2)	H14A—C14—H14C	109.5
C7—C8—Cl1	119.34 (17)	H14B—C14—H14C	109.5
C9—C8—Cl1	119.48 (17)	C1—N1—C2	111.59 (17)
C8—C9—C10	118.6 (2)	C1—N2—H2A	120.0
C8—C9—H9	120.7	C1—N2—H2B	120.0
C10—C9—H9	120.7	H2A—N2—H2B	120.0
			
C3—S1—C1—N1	0.05 (16)	C6—C7—C8—Cl1	179.63 (17)
C3—S1—C1—N2	−178.88 (18)	C7—C8—C9—C10	−1.1 (3)
N1—C2—C3—C4	175.9 (2)	Cl1—C8—C9—C10	179.78 (16)
C11—C2—C3—C4	−3.8 (4)	C8—C9—C10—C5	1.4 (3)
N1—C2—C3—S1	−1.0 (2)	C6—C5—C10—C9	−1.0 (3)
C11—C2—C3—S1	179.30 (18)	C4—C5—C10—C9	179.46 (19)
C1—S1—C3—C2	0.54 (16)	C3—C2—C11—C14	−10.1 (3)
C1—S1—C3—C4	−177.02 (16)	N1—C2—C11—C14	170.24 (19)
C2—C3—C4—C5	96.2 (3)	C3—C2—C11—C13	110.3 (2)
S1—C3—C4—C5	−87.0 (2)	N1—C2—C11—C13	−69.4 (2)
C3—C4—C5—C6	−6.9 (3)	C3—C2—C11—C12	−130.7 (2)
C3—C4—C5—C10	172.63 (18)	N1—C2—C11—C12	49.7 (2)
C10—C5—C6—C7	0.4 (3)	N2—C1—N1—C2	178.26 (18)
C4—C5—C6—C7	179.9 (2)	S1—C1—N1—C2	−0.6 (2)
C5—C6—C7—C8	−0.2 (3)	C3—C2—N1—C1	1.1 (3)
C6—C7—C8—C9	0.5 (3)	C11—C2—N1—C1	−179.18 (17)

### Hydrogen-bond geometry (Å, °)

**Table:**

*D*—H···*A*	*D*—H	H···*A*	*D*···*A*	*D*—H···*A*
N2—H2A···N1^i^	0.88	2.24	3.032 (2)	150

Symmetry codes: (i) −*x*+1, −*y*+2, −*z*.
